# Effect of the surface coverage of an alkyl carboxylic acid monolayer on waterborne and cellular uptake behaviors for silicon quantum dots

**DOI:** 10.1038/s41598-022-21698-z

**Published:** 2022-10-14

**Authors:** Naoto Shirahata

**Affiliations:** 1 International Center for Materials Nanoarchitectonics (MANA), National Institute for Materials Science (NIMS), 1-1 Namiki, Tsukuba, Ibaraki 305-0044 Japan; 2 Graduate School of Chemical Sciences and Engineering, Hokkaido University, Kita 13, Nishi 8, Kita-ku, Sapporo, 060-0814 Japan; 3 Department of Physics, Chuo University, 1-13-27 Kasuga, Bunkyo, Tokyo , 112-8551 Japan

**Keywords:** Materials science, Nanoscale materials, Nanoparticles

## Abstract

This article reports the development of highly waterborne silicon quantum dots (Si QDs) terminated with a reactive group for grafting of biomolecules. Hydrogen-terminated QDs were prepared by thermal disproportionation of amorphous hydrogen silsesquioxane derived from triethoxysilane followed by hydrofluoric etching. Next, the hydrogenated Si surfaces were exposed to 10-undecenoic acid at different temperatures in Ar atmosphere, yielding the termination of the QDs with a carboxyl group. The thermal hydrosilylation of 10-undecenoic acid yielded the termination of the QDs with a carboxyl group. An increase in molecular coverage of an undecanoic acid (UA) monolayer resulted in both the enhanced increase of zeta-potential in a negative direction for a greater water-dispersity and the increase of absolute quantum yield (QY) of photoluminescence (PL). PLQY improved for ~ 1% to 26% with increasing UA coverage. We assessed the molecular interaction between the UA-SiQDs and HeLa cells by means of cellular uptake experiments using the QDs with different UA coverages. Results showed that the QDs with the highest dispersity in water were not internalized in the cells under confocal fluorescence microscopic observation. In contrast, the QDs with lower coverage of UA monolayer were internalized by endocytosis when incubated with HeLa cells. This contrasting observation opens the possibility of successfully preparing carboxy-capped SiQDs that do not allow cellular uptake but are targeted to specific cells by appropriate conjugation with biomolecules.

## Introduction

Growing minimally invasive medicine have spurred explorations in fluorescent probes that could be excited with red-to-near-infrared (NIR) light for diagnostic and therapeutic imaging^1–5^. Colloidal quantum dots (QDs) of semiconductors as one of the probes have attracted attention due to their unique optical properties including a spectral tunability of optical absorbance and emission bands, a high photostability, and a broad wavelength range for photoexcitation. The NIR spectral region used for biological imaging is segmented into the NIR-I (λ = 700–950 nm), NIR-IIa (λ = 1000–1350 nm) and NIR-IIb (λ = 1500–1800 nm) biological windows. There are four classes of Cd–, Pb– and Hg–free colloidal QDs emitting the red-to-NIR light that include Group IV (C, Si, Ge, Ge_1-x_Sn_x_ alloy, and SiGeSn alloy)^6–14^, Group III-V (InN, InP, InSb, InAs and InAsSb alloy)^15–22^, Group I-VI (Ag_2_S, Ag_2_Se and Ag_2_Te)^23–28^ and Group I-III-VI (CuInS_2_, CuInSe_2_, AgInSe_2_ and ZnS-AgInS_2_)^29–33^ semiconductors. Other way for emerging red-to-NIR emission is the use of rare-earth doped nanocrystals^34–36^, or Pb-free perovskite nanocrystals such as CeSnI_3_, Sb-doped Cs_2_SnCl_4_Br_2_, and lanthanide-doped CsMnBr_3_^37–40^. Most of these QDs and nanocrystals exhibit the downshifting luminescence, a single-photon process that converts absorbed high-energy photons to low-energy ones while those with excitation maxima in the short-wavelength region are efficiently photoexcited for red-to-NIR emission via upconversion techniques^41–44^. Nevertheless, the red-to-NIR-light emitter used in the clinical spot for surgical medical treatments is limited to indocyanine green (ICG). In general, semiconductor QDs are chemically robust and more resistant to light fading than conventional organic dyes^45^.

Silicon (Si) QD has attracted attention since 2004 in which the conjugation process was reported for labeling of its nanoparticles to an oligonucleotide^46^. SiQDs are known to have lower inherent toxicity than other ones, as evidenced by in-vitro studies in tissues and in-vivo studies in mice^47^. Their superior photoluminescence (PL) properties include the narrow red-to-NIR PL spectra having no long emission tails^48^, continuously tunable over the 650–1000 nm wavelength range^48,49^, and decay times on the scale of 100 μsec^48^. Furthermore, by taking advantage of multiphoton absorption or fluorescence resonance energy transfer (FRET) processes, SiQDs emit the red-to-NIR light through the sequential absorption of photons from the red-to-NIR light^41,48,50,51^.

Now, the surfaces of red-to-NIR light-emitting SiQDs that have been generated in hydrofluoric acid due to liberation from oxidized Si are terminated with hydrogen atoms. So, the resultant SiQDs are not dispersible in biological buffers without further modification. Generally, two methods have been used to make the hydrophobic QDs dispersible in the buffer: (1) coating of assemblies of the QDs with amphiphilic biocompatible polymers and (2) termination of the individual QDs with polar functional group. In the first approach, low hydrophilic QDs are encapsuled with an amphiphilic biocompatible polymer^47,48^. For the second approach, the terminal hydrogen atoms are replaced by hydrocarbon chain with polar functional group at the end such as carboxylic acid or amine. Due to the terminal carboxyl or amino group, the water-dispersity is given to individual QDs^52^. The QDs with those functional groups could be conjugated with biomolecules using traditional method. For example, SiQDs terminated with carboxyl groups, known to be lesser toxicity than amine^53^, react with alkylamines functionalized with biomolecules using carbodiimide chemistry, giving a targetability for the specific cell to the QDs. In semiconductor QDs except for Si, the remaining carboxyl groups on the surface are passivated by reacting with amino alcohol to yield a hydroxyl group. However, fluorescence of the SiQD is known to quenched by addition of primary amine^54^, preventing the use of amino alcohols. Thus, the challenging aspect of surface engineering is how to prepare carboxy-terminated SiQD that do not allow cellular uptake and high PLQY is preserved at the same time.

In the present study, the interaction between the waterborne SiQDs and the HeLa cell was investigated for difference in molecular coverage of 10-undecanoic acid (UA) monolayer covalently bonded onto the QD. The results shown provides a better understanding of surface chemistry to improve the dispersity of SiQD in water.

## Experimental procedure

### Reagents and chemicals

All reagents and chemicals were used as received. Triethoxysilane (TES) and 10-undecenoic acid were purchased from Tokyo Chemical Industry (TCI) Co., Ltd. Hydrochloric acid (HCl, 49% aqueous, electronic grade) and hydrofluoric acid (HF, 46–51% aqueous solution with metal impurity of less than 100 ppm) were purchased from Kanto Reagents. Milli-Q water was purified by Sartorius Arium 611UV water purification system. Other solvents such as ethanol, acetonitrile, and dichloromethane were purchased from Wako Chemicals, Japan. For bimodal imaging observations, HeLa cell lines were obtained from the Riken Bio-Resource Center (Tsukuba, Japan). Phosphate Buffered Saline (PBS) was purchased from Wako Chemicals, Japan. Minimum essential media (MEM) with phenol red, Penicilin Streptomycin, 0.05% Trypsin-EDTA, 4% Paraformaldehyde Phosphate Buffer Solution (PFA) and DAPI staining solution were purchased from Thermo Fisher Scientific, USA. Fatal Bovine Serum (FBS) was purchased from Sigma-Aldrich, USA.

### Preparation of UA-terminated SiQDs

**Figure 1:**
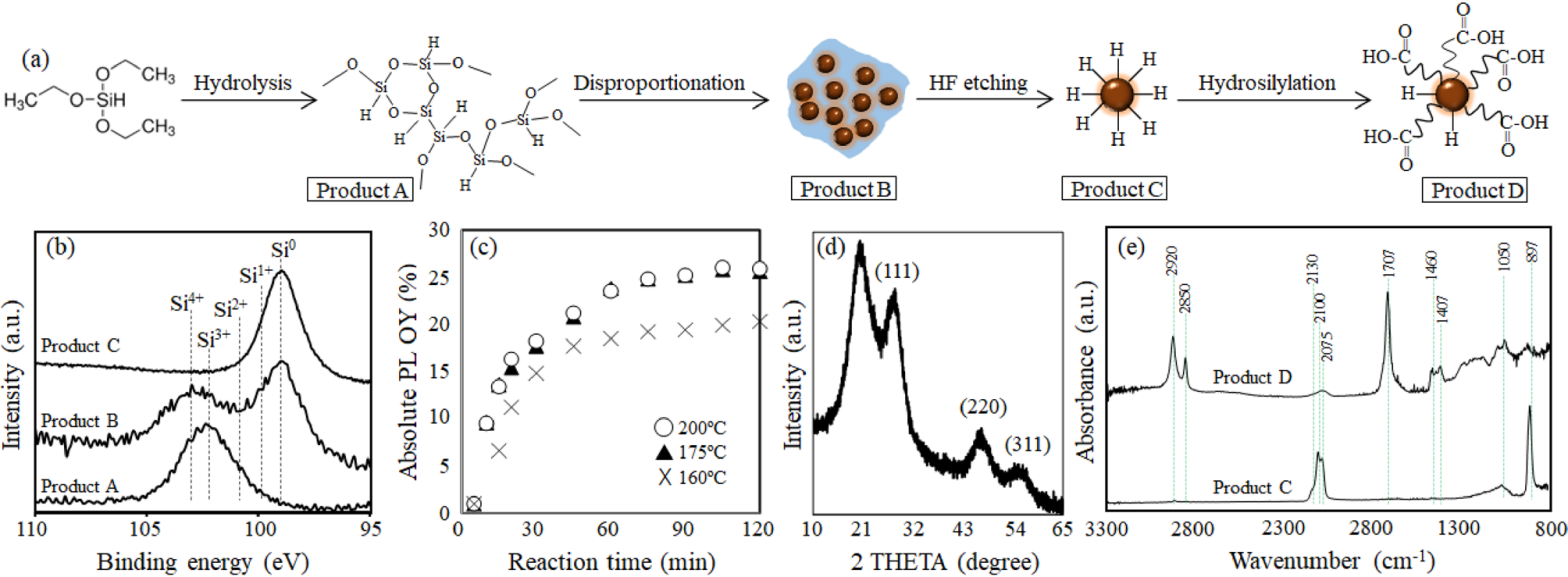
(**a**) Scheme of colloidal SiQD terminated with undecane monolayer. (**b**) XPS Si 2p spectra of the Products A, B and C. (**c**) Absolute values of PLQY of SiQD as a function of reaction temperature for hydrosilylation of 10-undecenoic acid. (**d**) A typical XRD pattern of undecanoic acid terminated SiQD. (**e**) ATR-FTIR spectra of the Products C and D.

The UA-SiQDs were prepared by a four-step method which consists of hydrolysis and condensation of TES followed by heat treatment, HF-etching and hydrosilylation of 10-undecanoic acid as shown in Fig. 1a. The first three-steps followed the previous method^55^. In a typical, 16 mL of TES was hydrolyzed at room temperature in Ar atmosphere by dripping aqueous solution of HCl having a pH 3. The resultant gel-like product was filtered followed by washed with Milli-Q water until the pH become 7. This precipitate was dried overnight under vacuum, yielding a white fine powder.

The white powder was subjected to heat treatment into a vacuum furnace in a quartz crucible. First, the inside of the crucible was evacuated by a vacuum pump until 5 Pa and then refilled with 5%-H_2_/95%-Ar gas. The gas purge was repeated three times. The powder was heated at 1050ºC for 2 h to yield a brown powder. 300 mg of brown powder was grinded in an agate mortar then subjected to HF etching by stirring in 8 mL of ethanol and 16 mL of HF for 50 min. After stirring, solution was centrifuged at 15,000 rpm for 5 min at 10 °C in ethanol, acetonitrile, and dichloromethane in this order. The dichloromethane became turbid because HF-etched product was not dispersed in any solvents.

Prior to hydrosilylation, 10-undecanoic acid was degassed at the temperature of 70 °C and the pressure of 30 Pa or lower. The dichloromethane solution of HF-etched powder was transferred to a two-neck round bottom flask containing 15 mL of the degassed 10-undecanoic acid. The dichloromethane was completely removed by evacuation of the solution at room temperature using a vacuum pump. Then, the solution was heated in Ar atmosphere to the temperature of 160–200 °C within 3 min. The duration of time for reaction was varied between 0 and 3 h. After hydrosilylation, solution was split into four 30 mL centrifuge tubes and the rest of the tubes were filled with ethanol. Centrifugation was done at 15,000 rpm for 5 min at 10 °C. Precipitated product was dispersed in 4 mL of ethanol and hexane as an antisolvent. The centrifugation for washing the QDs was repeated at least five times. The washed QDs were dispersed in water of pH 8.3 or 7. The dispersity in water of pH 7 was obtained by gradual exchange of the solvent from ethanol to water. Specifically, the ethanol solution of UA-SiQDs was filled in a vial and same amount of water was added in. Then, this solution was subjected to vacuum drying until the volume becomes its half.

### Characterization

Crystallinity and crystal structure of the sample were determined by X-ray powder diffractometer (XRD, Miniflex 600, Rigaku, Japan) with Cu-K_α_ radiation (λ = 0.15406 nm). Ethanol solution of the QDs was drop-casted onto a reflection free sample holder of a single crystal Si. XRD pattern was obtained by scan with 0.2 step size and 2°/min scan speed. The oxidation sates of the samples were determined by X-ray photoelectron spectroscopic (XPS; Shimadzu, ESCA3400) study using MgKα (E = 1253.6 eV) radiation. The binding energy scale was calibrated to provide Au4f_7/2_ = 83.9 eV and Cu2p_3/2_ = 932.8 eV. The X-ray source was operated at 10 mA and 12 kV. The core-level signals were obtained at a photoelectron take-off angle of 90° (with respect to the sample surface). Data acquisition and processing were performed by a SUN Microsystems ULTRA 5 computer, using the VISION 2.0 processing package. The BE scales were referenced to 285.0 eV as determined by the locations of the maximum peaks on the C1s spectra of hydrocarbon, associated with an adventitious contamination. Attenuated Fourier Transform Infrared Spectroscopy (ATR-FTIR, Jasco FTIR-4100, Japan) was used to determine the chemical bonding states of the sample surface. Zeta potentials of the samples dispered in water of pH 7 were measured by Malvern Zetasizer Nano-Z equipment. The sample in powder form was analyzed under N_2_ flow in the 800–3300 cm^−1^ range. Thermogravimetric (TG) analysis was performed for calculation of surface coverage of UA monolayers. 4.0 mg of the sample in powder form was heated up to 650 °C with 10 °C/min heating rate under Ar atmosphere in alumina crucibles and mass loss was recorded. By using this data, surface coverage was calculated by the previously reported method^56^.

Photoluminescence (PL) properties of the samples were measured by a modular double grating Czerny-Turner monochromator with an iHR 320 emission monochromator (1200 lines/mm of gratings) coupled to a photodetector on a NanoLog Horiba Jobin Yvon spectrofluorometer with a 450 W Xenon arc lamp. The absolute PL quantum yields (QYs) of the samples in liquid form were measured at room temperature using the C9920-02 QY measurement system from Hamamatsu Photonics Co., Ltd. with a 150 W xenon lamp coupled to a monochromator for wavelength discrimination, an integrating sphere as a sample chamber, and a multichannel analyzer for signal detection. The sample in a liquid form was subjected to a UV–vis spectrophotometer (JASCO V-650, Japan) for optical absorption measurement.

### In-vitro fluorescence imaging

A 500 µL of HeLa cells in MEM solution at a cell density of 35,000 cells/mL were seeded into 4-chamber 35 mm glass bottom dishes. After 24 h incubation, cell culture media was changed to new culture medium containing 100 µg/mL of the SiQD-MEM solution. After 1 h incubation, cells were washed thrice with PBS and then fixed in 3.7% paraformaldehyde solution. Prepared dishes were examined under a confocal laser scanning fluorescence microscope (SP5, Leica Microsystems, Germany) under a 405 nm laser. Control cells without UA-SiQDs treatment were also observed to eliminate the errors by autofluorescence.

## Results and discussion

**Figure 2:**
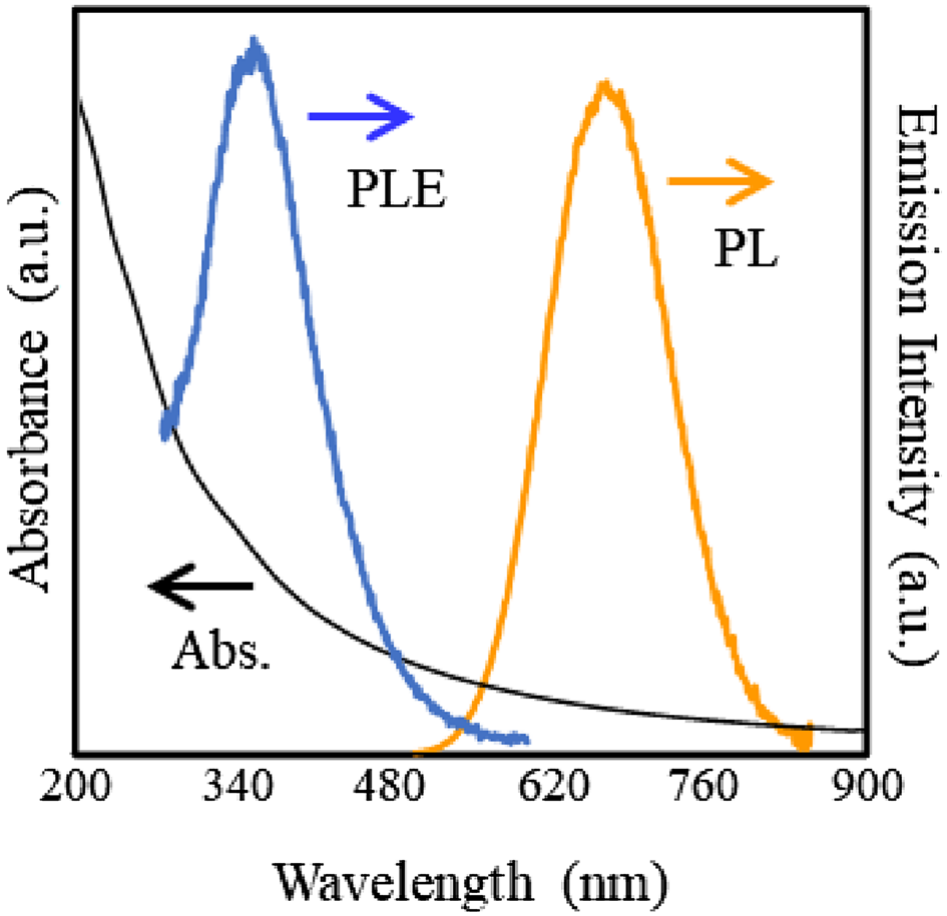
A typical optical absorption, excitation and emission spectra of the Product E.

Preparation of the waterborne SiQDs is represented schematically in Fig. 1a. First, the hydrolysis and condensation of TES molecules were performed at pH 3 to prepare the white-color powder (Product A). In Fig. 1b, the XPS Si2p spectrum of the product A shows a broad, single peak centered at 102.3 eV. The chemical shift of 3.0 eV with respect to that of the Si substrate (i.e., 99.3 eV for Si^0^) corresponded to the Si^3+^ chemical state which peaks at 101.8–102.7 eV^57–59^, indicating the formation of hydrogen silsesquioxane. On the other hand, the full width at half maximum (FWHM) of the peak was slightly broader than the ideal value for a single component of Si^3+^ (i.e., 660–760 meV), suggesting that other oxidation states such as Si^4+^ might be contained as a minor constituent in the Product A. The white-color powder of (HSiO_1.5_)_n_ was thermally disproportionated into two oxidation chemical states of Si as Si^0^ and Si^4+^^60^, yielding a dark brown powder (Product B). As expected, the XPS Si2p spectrum was split to two peaks centered at 99.3 eV for Si^0^ and 103.6 eV for Si^4+^. This indicated that the Product B consists of SiQDs dispersed in SiO_x_ matrix. The SiQDs were liberated from the oxide matrix by HF etching as evidenced in the XPS Si_2_p spectrum (Product C). ATR-FTIR spectrum of the Product C is shown in Fig. 1e. The absorption peak centered at 897 cm^−1^ represented SiH_3_ degenerate deformation and SiH_2_ scissoring modes^61^. A broad absorption peak centered at near 2100 cm^−1^ was the split in three vibration modes at 2075 cm^−1^ for monohydride (≡Si─H), 2100 cm^−1^ for dihydride (═SiH_2_) and 2130 cm^−1^ for trihydride (─SiH_3_) bonds on SiQD^62^. A low intensity peak centered at 1050 cm^−1^ appeared possibly due to the remained intermediate oxidation state of Si (Si^1+^), consistent with the XPS Si_2_p spectrum of the Product C. Thermal hydrosilylation of 10-undecenoic acid was performed to prepare the SiQDs terminated with undecanoic acid monolayers (UA-SiQDs, Product D). The reaction temperature was varied between 160 and 200 °C. Figure 1c shows the changes in PLQY of UA-SiQDs prepared at different reaction times of 160–200 °C. The PLQY of H-SiQD was as low as 1% while increased with reaction time and became constant after 2 h. Furthermore, high PLQY was obtained at reaction temperatures above 175 °C. 26% of PLQY is the highest value ever obtained for carboxy-terminated SiQDs. The relationship between PLQY, reaction time and temperature was found to be independent of the diameter of the H-SiQDs. PLQY of the SiQD in solid form was close to that of the counterpart in liquid form (see Figure S1, supporting Information). Figure 1d shows a typical XRD pattern of the UA-SiQD prepared at 175 °C for 2 h. The diffraction peaks at 2θ = 28, 47 and 56° corresponded to (111), (220) and (311) planes, indicating the formation of a diamond cubic structure. A broad peak centered at ~ 20° appeared due to the presence of the UA monolayer; the absence of this peak in the H-SiQD supported this identification. The average diameter of UA-SiQDs estimated by Scherrer’s spectral width analysis was about 1.85 nm. In this study, the sample was not characterized with other size measurement techniques such as a small-angle X-ray scattering (SAXS) or a transmission electron microscopy (TEM) because the diameter estimated by XRD peak broadening analysis with Scherrer equation corresponds with the theoretical and experimental values obtained from the effective mass approximation model and SAXS under the limited conditions that diamond cubic SiQDs of d < 2 nm are prepared by thermal disproportionation of hydrogen silsesquioxane^55,63^. Surface characteristics were investigated by ATR-FTIR for the UA-SiQDs as shown in Fig. 1e. It was seen that peaks at 1400–1450 cm^−1^, 2850 and 2920 cm^−1^, 1707 cm^−1^, and 1210–1320 cm^−1^ belonging to Si–CH_2_ scissoring mode, the symmetric and asymmetric CH_2_ vibrational modes, and the stretching C=O vibrational mode and C–O stretching mode, respectively, appeared after hydrosilylation. There were peaks at 2100 cm^−1^ and 897 cm^−1^ belonging to Si–H bondings while a tiny peak at 1050 cm^−1^ corresponding to oxidation state of Si was remained. It should be noted that peak at 2100 cm^−1^ could still be observed after hydrosilylation indicating that not all of the Si atoms on the surface could be terminated by UA monolayers (see the spectrum of Product D). Figure 2 shows UV–vis absorption, PLE and PL spectra of a typical sample of UA-SiQDs that are highly dispersed in Milli-Q water. The absorption spectrum exhibits a long absorption tail possibly reflecting the bulk band structure. The PL spectrum shows a peak at 685 nm, which corresponds to a SiQD with an average diameter of 1.8 nm based on the correlation between PL photon energy and QD size established in previous theoretical and experimetanl studies^55,63,64^.

**Figure 3:**
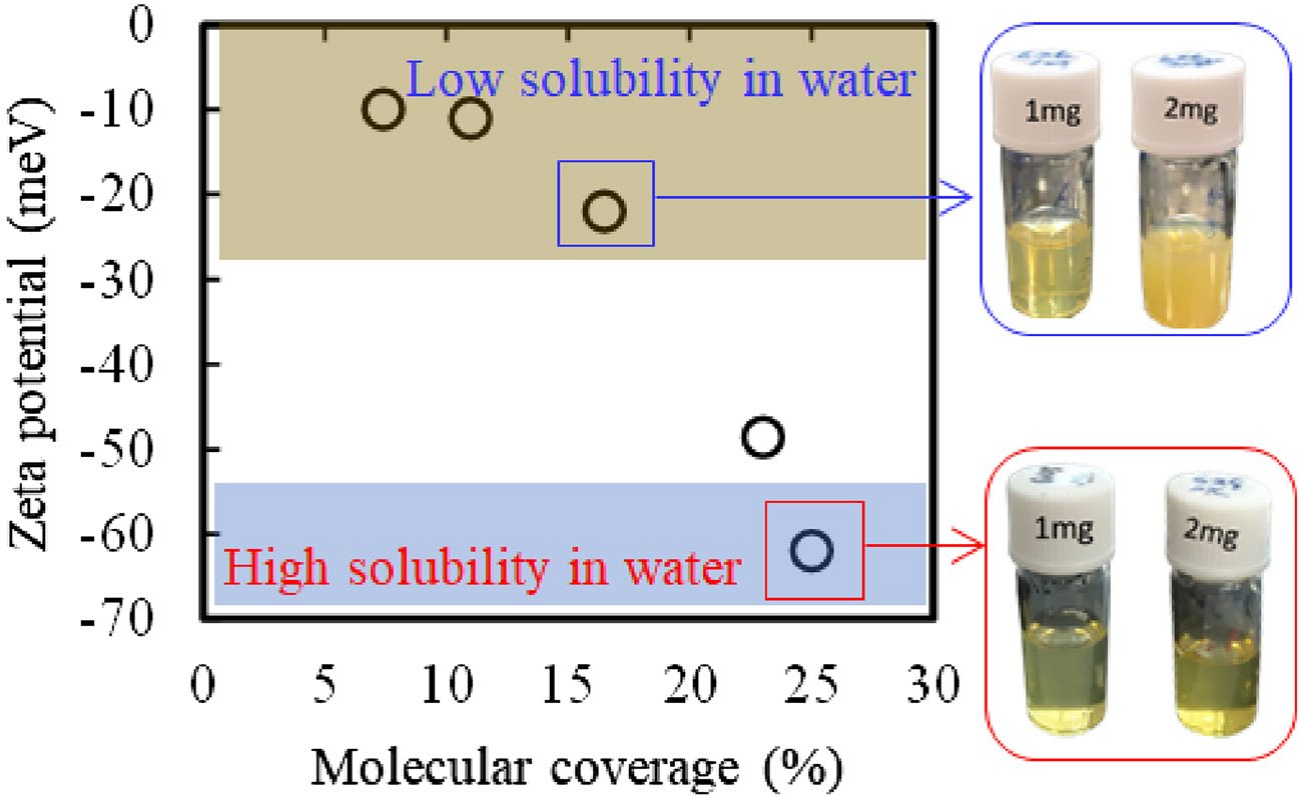
Changes in zeta-potential of SiQDs with increasing molecular coverage of 10-undecanoic acid monolayer. Digital photographs show the difference in transparency of SiQDs with different monolayer coverage dispersed in milli-Q water at 1.0 mg/mL and 2.0 mg/mL, respectively.

To date, surface fuctionalization of SiQD with various molecule ended with polar functional group has been investigated for giving a dispersity in water or biological buffers^64–69^, but the relationship between the monomolecular coverage, dispersity in aqueous media, and PL performance has not yet been reported despite the fact that achieving both of these three parameters is an important for bioimaging agents that work without aggregation or nonspecific cellular adsorption. To reveal the relationship, some of the samples shown in Fig. 1c were subjected to thermogravimeter-differential thermal analyzer, zeta-potential analyzer and absolute PLQY spectrofluorometer. In the TG analysis, the UA molecular coverages were estimated on the assumption that the lost of weight in Ar atmosphere belongs to all the UA molecules (see Figure S2, Supporting Information). Using the measured weight reduction and theoretical value of surface atom number belonging to 1.836 nm diameter with T_d_ symmetry, formulated as Si_147_H_100_^70^, the values of molecular coverage were calculated for each sample. The results are plotted in Fig. 3. The best value (~ 62 meV) of zeta-potential in Milli-Q water was obtained for the QD with UA coverage of 25% and this was the record value among the reported ones of the carboxy-terminated nanoparticles^71^. The zeta-potential continued to decrease with decreasing UA coverage and was found to be almost saturated at − 10 meV when the molecular coverage was below 11%.

**Figure 4:**
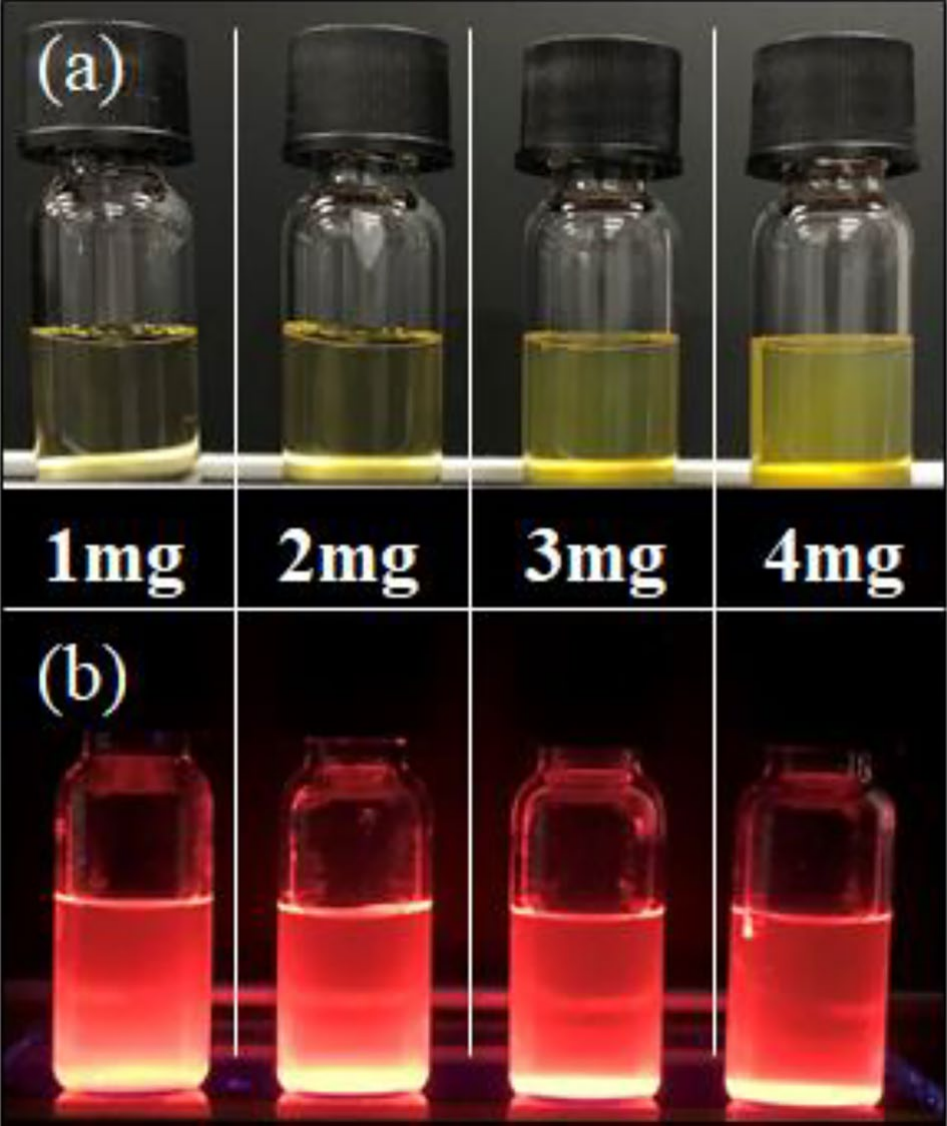
Digital photographs of milli-Q water containing 10-undecanoic-acid-terminated SiQDs at different concentrations under room light irradiation (upper line) and 365 nm UV light irradiation (lower line), respectively.

The carboxylated QDs were dispersible in borate buffer of pH 8.3 to become transparent pale-yellow solutions regardless of UA coverage because of deprotonation of terminal carboxylic acid under alkaline condition, whereas dispersity in milli-Q water (pH 7) was dependent of molecular coverage. For the concentration of 1.0 mg/mL, the aqueous solution of QDs with 25% UA coverage was transparent and pale-yellow, even when the concentration was doubled as evidenced in the digital photographs (see Fig. 3). However, aqueous solutions of 1.0 mg/mL QD with less than 15% molecular coverage were slightly turbid and became more turbid when the concentration was doubled (see the photos in Fig. 3). This difference in dispersity in milli-Q water was likely due to the difference in the amount of carboxyl group. As evidenced in Fig. 4, the transparency of the QD sample with 25% UA coverage did not change visually when the concentration in Milli-Q water was tripled and dropped slightly at quadrupling. The fluorescence images in the lower part of Fig. 4 were obtained under irradiation of a hand-held 365 nm light. The sample of 25% UA coverage shows a strong orange-red visible emissions, with PLQY of 26%, whereas the sample with 11% molecular coverage showed a PLQY of 16%, suggesting the improved PLQY with increasing molecular coverage. This increasing trend of PLQY with raise in UA monomolecular coverage was interesting observation result. Because the effective role of surface monolayer on improved PLQY has been reported for various semiconductor QDs^72–74^, but the molecular coverage as one of the parameters is not yet reported. For the case of SiQD, alkyl monolayers behave as an anchor that suppresses the amorphization of the outermost layer of SiQD, resulting in decrease of nonradiative recombination channels to improve PLQY^75^. According to this context, the 11% UA coverage might be too low to work as an anchor whereas the surface amorphization was possibly suppressed for the 26% UA-coveraged QD. A long-term PL stability test taken at 37 °C showed that no change in the spectral peak shape and PLQY for at least 15 days while PL photon energy increased by 35 meV (see Figure S3, Supporting Information). The small PL spectral shift to a longer wavelength side could be due to the decrease of QD size with time dependent oxidation, provoking an increase in the fundamental energy gap based on the quantum confinement effect.

**Figure 5:**
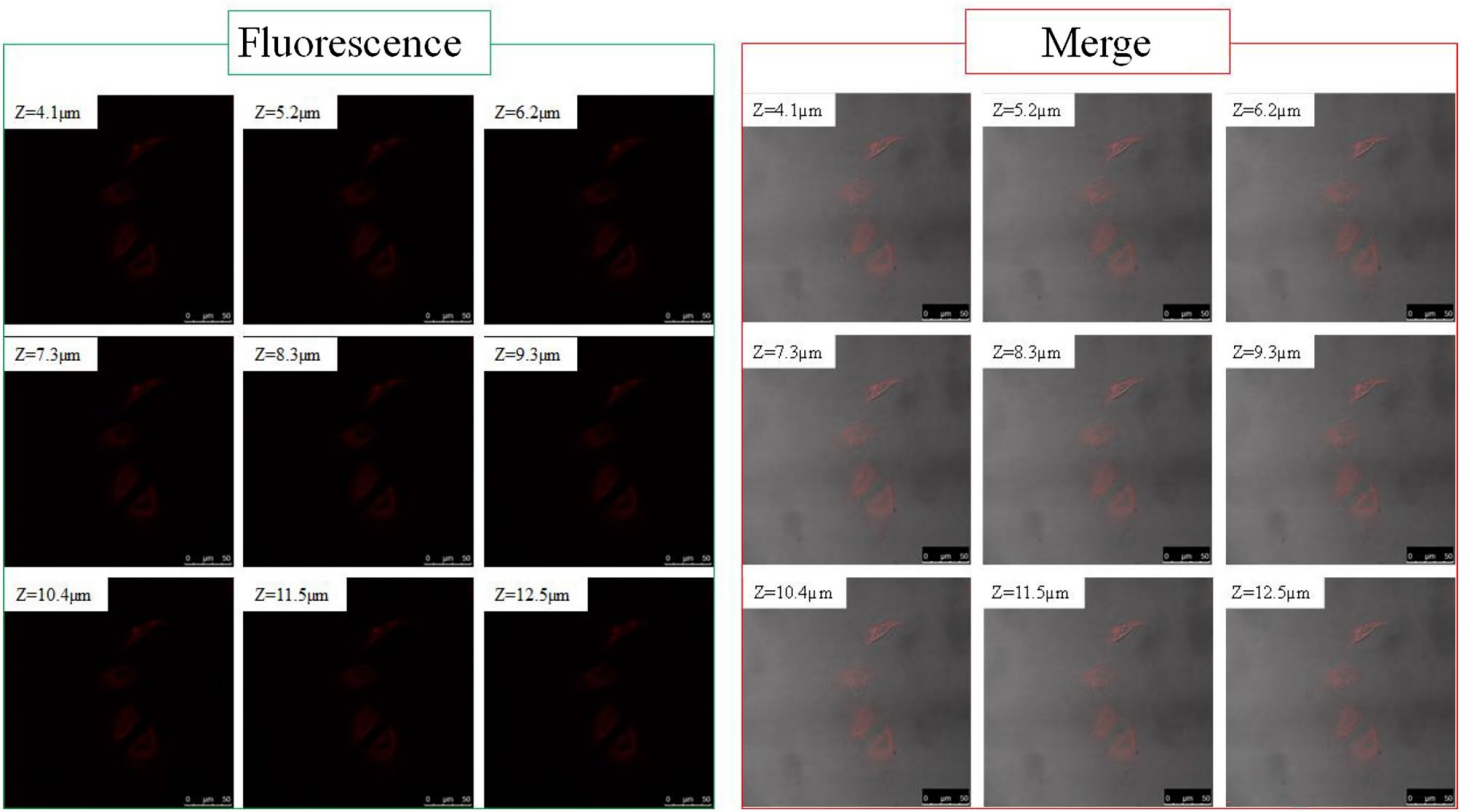
HeLa cellular uptake of UA-SiQDs with 11%-UA-coverage under confocal microscopy (Left: fluorescence. Right: overlap images). Slice scan of HeLa cells under confocal microscopy. Bar shows 50 μm.

**Figure 6:**
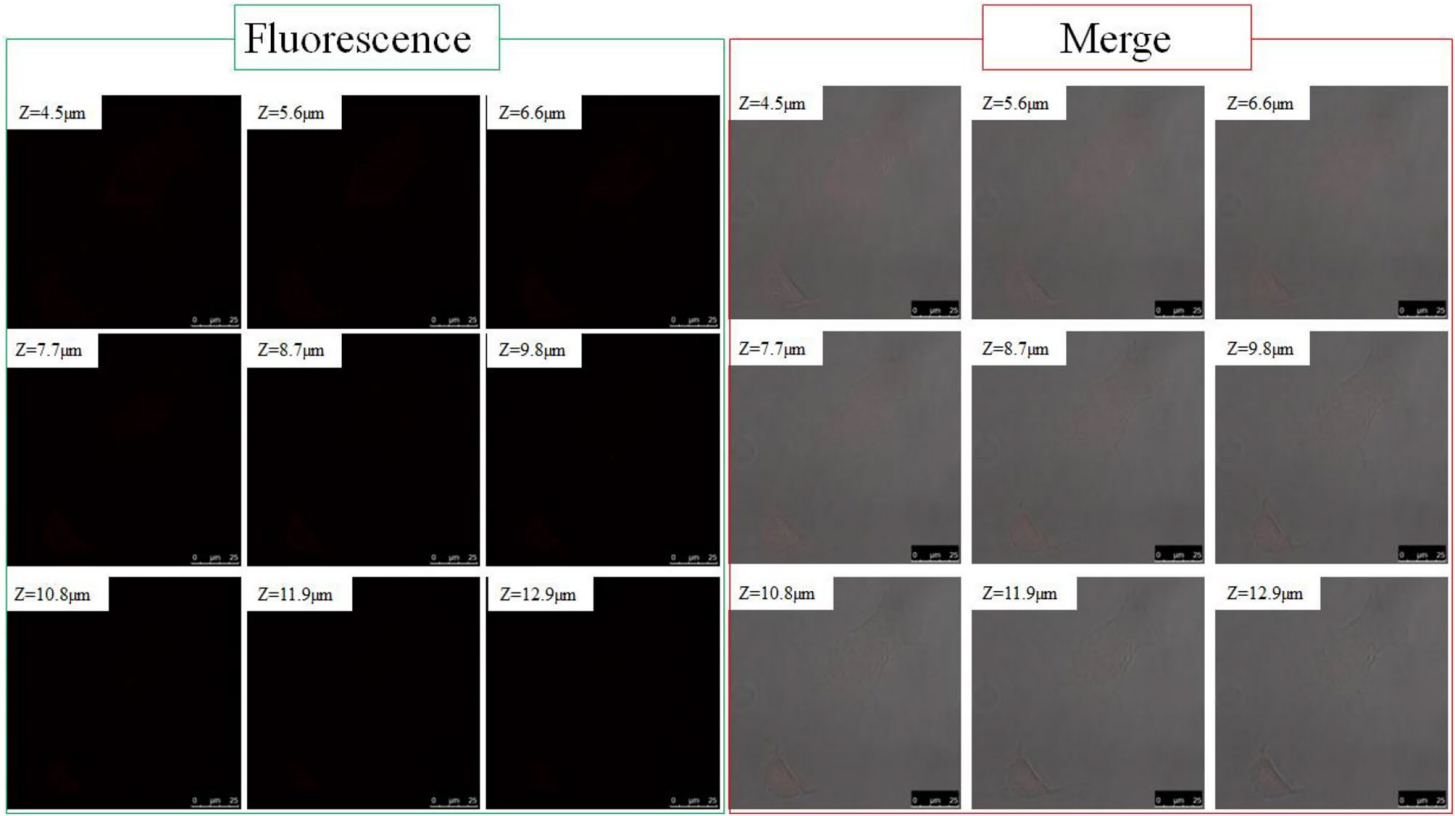
HeLa cellular uptake of UA-SiQDs with 25%-UA-coverage under confocal microscopy (Left: fluorescence. Right: overlap images). Slice scan of HeLa cells under confocal microscopy. Bar shows 25 μm.

Fluorescence detection of cells labeled with QDs is one of non-invasive techniques since the QDs are internalized based on the ability of cells to endocytose. The surface of QDs terminated with reactive groups such as carboxylic acid or amine can be further functionalized, followed by conjugation with biomolecules to provide targeting properties to cancer cells, but the endocytic uptake of QDs by normal cell should be blocked for specfic adsoption. Generally, the electronically neutral surface, which is realized by termination of hydroxyl group, is known to suppress the endocytosis. As a new strategy for the suppression, this paper reveals that a high coverage of carboxylic acid monolayer would prevent QDs from being internalized or nonspecifically adsorbed. To test, HeLa cell was incubated for 1 h at 37 °C with the 1.0 mg/mL QDs having 11%- or 25%-UA coverage and then washed to remove excess QDs. Figure 5 shows the typical conforcal fluorescence microscopic images of HeLa cells labelded with the QDs of 11%-UA coverage. The luminescence was slightly visible in the fluorescence images (see the nine photos on the left), but not enough to identify the QDs internalized in the cells. In contrast, the merged images clealry show the QDs internalized in the cells as preudocolored with red (see the nine photos on the right). A consecutive sectional scan of HeLa cells along the Z-direction confirmed the cellular uptake of the QDs. The QDs were distributed throughout the cytosol and did not enter the cell nucleus (see also Figures S4 and 5, Supporting Information). Figure S6 shows the result of confocal microscopic observation of HeLa cells incubated at 4 °C for 1 h together with 200 μg/mL SiQDs of 11%-UA coverage. Since a cellular uptake is dependent of energy, the endocytosis is negligible at 4 °C.In Figure S6, no fluorescence signals were obtained even in merged image, suggesting that the uptake of the QDs by HeLa cells observed in Fig. 5 is an energy-dependent process such as endocytic pathways although further study is needed to identificate the QD uptak pathway. Figure 6 shows the confocal fluorescence microscopic images of HeLa cells labeled with the QDs of 25%-UA coverage. There was no fluorescence signal originating from the QDs when the UA-SiQDs with the 25% coverage were incubated with the cells (see Fig. 5b). Even if there were, it would be in such small quantities that it would be impossible to visualize under the conventional fluorescence microscope. This contrasting observation results might be a result of the QDs aggregated due to low molecular coverage which are uptaken by the cell when QDs of 11%-UA coverage was incubated with the cells. This further indicates that the QDs were internalized by endocytosis and not as a result of compromised permeability of the plasma membrane, as evidenced by no fluorescence signals from the HeLa cells incubated with QDs of 25% UA-coverage. This confocal microscopic study opens the possibility of successful preparation of SiQDs that are not internalized in normal cells but having a targetability to the specific cell.

## Conclusion

Waterborne SiQD was synthesized by hydrosilylation of 10-undecenoic acid on the hydrogen-terminated SiQD derived from the hydrolysis product of triethoxysilane. High monolayer coverage was achieved by increasing the reaction temperature of the hydrosilylation above 170 °C. As expected, the zeta-potential of the carboxylated SiQD surface was dependent of the monolayer coverage. SiQDs terminated with the monolayer coverage of more than 25% were dispersed highly in milli-Q water (pH 7) even at high SiQD concentration of 4.0 mg/mL. In contrast, monolayer coverage of 15% was too low to disperse SiQDs at concentrations above 1.0 mg/mL. The SiQDs with 25% monolayer coverage maintained a water-dispersity and stong intensity of PL after 15 days of extension. Differences in water dispersity at pH 7 based on surface monolayer coverage were found to affect the cellular uptake of SiQDs. The SiQDs with low monolayer coverage were internalized by endocytosis when incubated with HeLa cells at 37 °C whereas the cells incubated at 4 °C with UA-SiQDs did not internalize the QDs, suggesting that the QDs of low UA coverage took the QDs through energy-dependent process. As expected, the SiQDs with high monolayer coverage were not observed in HeLa cells even after incuvation at 37 °C. It is noted that high monolayer coverage is an important parameter to prepare a waterborne fluorescent QD having specific cell adsorption ability.

## Supplementary Information

Supplementary Information.

## Data Availability

The raw data and material used and analyzed in this study are available from the corresponding author on reasonable request.
