# Supplementary material for: Effect of the surface coverage of an alkyl carboxylic acid monolayer on waterborne and cellular uptake behaviors for silicon quantum dots
Source: Sci Rep. 2022 Oct 14;12:17211. doi: 10.1038/s41598-022-21698-z (PMC9568572; doi:10.1038/s41598-022-21698-z)
Supplement: Supplementary file 1 — Supplementary Information. [file 41598_2022_21698_MOESM1_ESM.docx]

Supplementary Information

Effect of the Surface Coverage of an Alkyl Carboxylic Acid Monolayer on Waterborne and Cellular Uptake Behaviors for Silicon Quantum Dots

Naoto Shirahata^1,2,3^

^1^International Center for Materials Nanoarchitectonics (MANA), National Institute for Materials Science (NIMS), 1-1 Namiki, Tsukuba, Ibaraki 305-0044, Japan

^2^Graduate School of Chemical Sciences and Engineering, Hokkaido University, Kita 13, Nishi 8, Kita-ku, Sapporo 060-0814, Japan

^3^Department of Physics, Chuo University, 1-13-27 Kasuga, Bunkyo, Tokyo 112-8551, Japan

Corresponding author: [SHIRAHATA.Naoto@nims.go.jp](mailto:SHIRAHATA.Naoto@nims.go.jp)

**Experimental section**

**Reagents**

The Hela cell line (RCB0007), purchased from RIKEN BioResource Center (Tsukuba, Japan).

**Calculation of UA monolayer coverage**

Surface coverage of the UA-SiQDs was calculated from the measured mass loss by Thermogravimetric Analysis (TGA) measurement. For the measurement, 3-4 mg SiQDs in powder form was put in alumina crucibles. The sample chamber was purged with Ar gas in several times to remove oxygen for precise measurement. Sample was heated with 10 °C min^-1^ rate up to 650 °C under Ar atmosphere. Surface coverage was calculated by the method reported by J. Mock *et al*.^1^ The specific procedure was shown below. The diameter of UA-SiQD was ~1.8 nm determined from the relationship between PL peak wavelength and QD size which is established in the 1.7-5.0 nm diameter range.^2^ According to S. Niaz *et al*., 1.836 nm T_d_ symmetry SiQD has the molecular formula of Si_147_H_100_.^3^ The total amount of 100 SiH surface groups per SiQD expressed $n_{SiH}$, is determined by the ratio of the sample weight at the end temperature $m_{end}$ to the molecular weight of the Si core $M_{Si}$. (Equation 1):

$$n_{SiH} \left[ mol \right]=\frac{m_{end}}{M_{Si}} \cdot100\quad\quad\left( 1 \right)$$

The amount of surface ligands $n_{Ligand}$ is determined by the ratio of the mass loss $\Delta m$ of the sample to the molar weight of the ligand $M_{Ligand}$. (Equation 2):

$$n_{Ligand} \left[ mol \right]=\frac{\Delta m}{M_{Ligand}}\quad\quad\left( 2 \right)$$

Finally, from equations (1) and (2), surface coverage can be obtained by Equation 3.

$$Surface coverage \left[ \% \right]=\frac{n_{Ligand}}{n_{SiH}}\cdot100\quad\quad\left( 3 \right)$$

**References**

1. Mock, J., Groß, E., Kloberg, M. J., Rieger, B., Becherer, M., Surface Engineering of Silicon Quantum Dots: Does the Ligand Length Impact the Optoelectronic Properties of Light-Emitting Diodes. Advanced Photonics Research 2 (2021) 2100083.

2. Shirahata, N., Nakamura, J., Inoue, J., Ghosh, B., Nemoto, K., Nemoto, Y., Takeguchi, M., Masuda, Y., Tanaka, M., Ozin, G. A., Emerging Atomic Energy Levels in Zero-Dimensional Silicon Quantum Dots. Nano Letters 2020, 20 (3), 1491-1498.

3. Niaz, S., Zdetsis, A. D., Comprehensive Ab Initio Study of Electronic, Optical, and Cohesive Properties of Silicon Quantum Dots of Various Morphologies and Sizes up to Infinity. The Journal of Physical Chemistry C 2016, 120 (20), 11288-11298.

**Results**


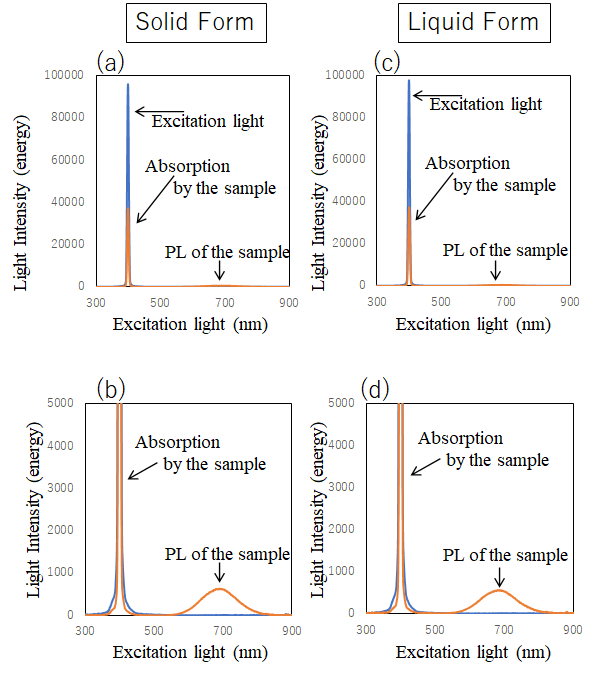


Figure S1. PLQY measurement profiles of the UA-SiQD in (a,b) solid form and (c,d) liquid form. The spectra of (b) and (d) are enlargement of a portion of the profiles (a) and (c), respectively. In the profiles, the blue-color peak shows the measured power of the incident light (λ= 400 nm) as excitation. A portion of the excitation light is absorbed by the QDs. The absorption amount as power is shown with orange-color peak at 400 nm. Based on the PLQY value, some of absorption is converted emission, resulting in PL peak at 685 nm. PLQY of the QD in solid form was measured to be 30%, while that of the same sample in liquid form was measured to be 26%.


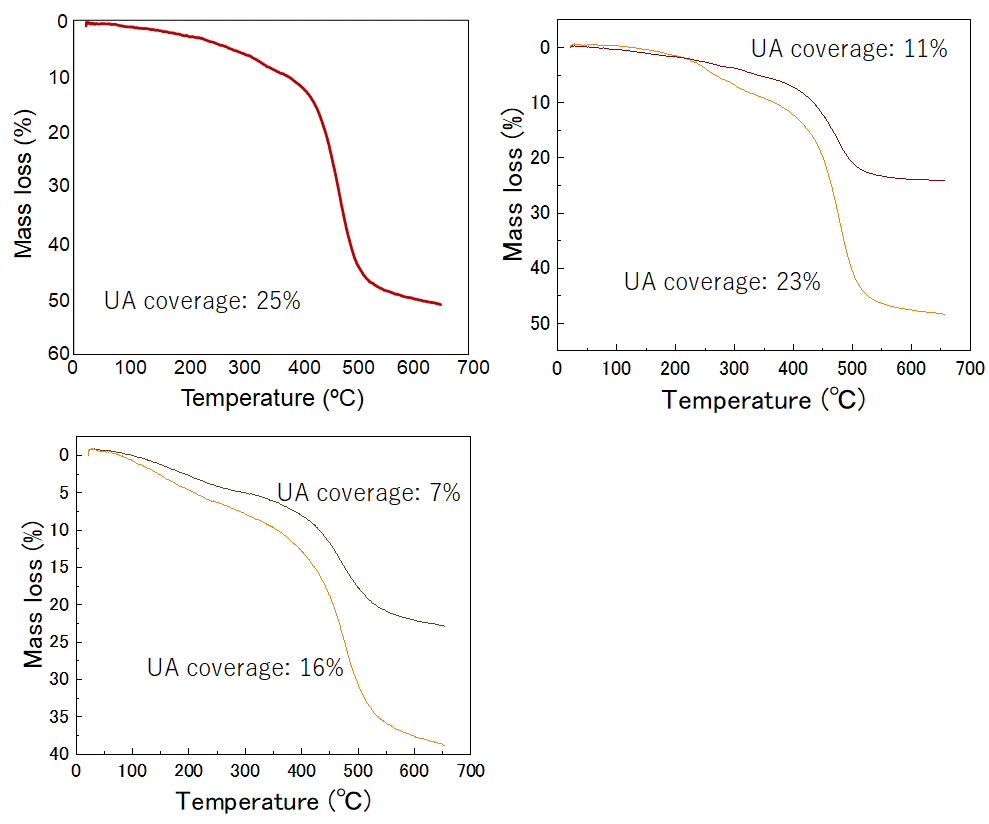


Figure S2. Mass change by heating UA-SiQDs obtained by TGA analysis. These TG curves were used to calculate the UA molecular coverages shown in Figure 3.


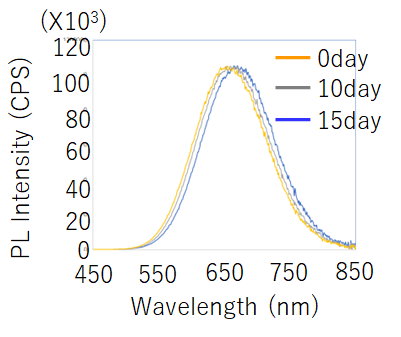


Figure S3. PL spectra of UA-SiQD (150 mg/mL) dispersed in milli-Q water at 37ºC after 10 and 15 days. The estimated PLQYs were 22% at 0 day and remained unchanged even after 15 days. This observation indicates that UA-SiQD were dispersed stably in milli-Q water at 37ºC for more than 2 weeks.


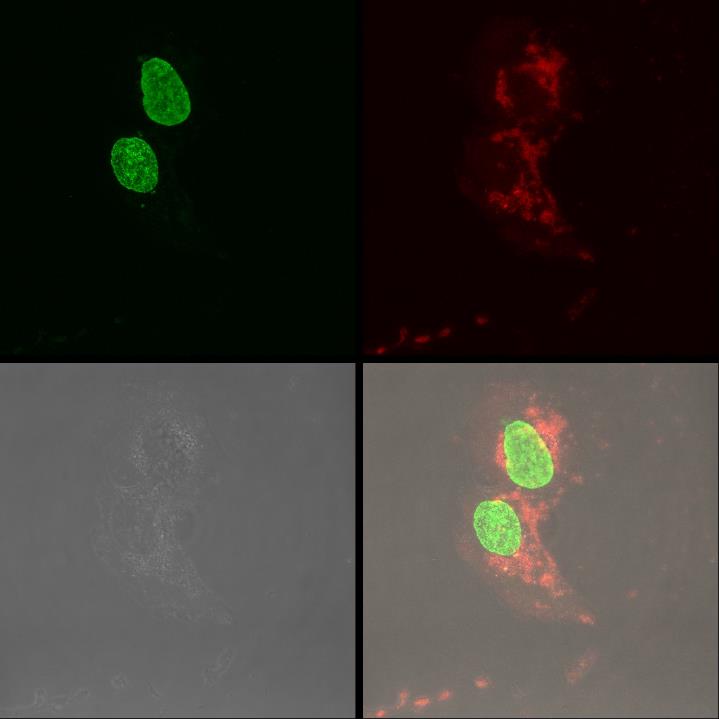


Figure S4. Fluorescence images of HeLa cells labeled with DAPI (upper left) and UA-SiQD with 16% molecular coverage (upper right). The DIC image of the same cell is shown in the lower left. The fluorescence image in the lower right shows the combined one between the two images presented in the upper line. The observation confirmed that the SiQDs were distributed throughout the cytosol and did not enter the cell nucleus.


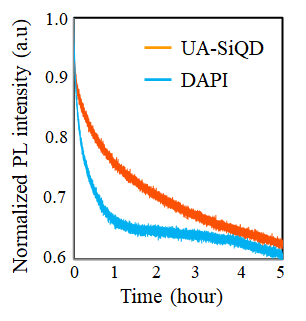


Figure S5. Stability of PL intensity with light-irradiation time for the UA-SiQD and DAPI. DAPI (4′,6-diamidino-2-phenylindole) is the commercial, the most famous and blue-fluorescent DNA stain. All the values of PL intensity were normalized to the values recorded at 0 min of the light irradiation. The values of PL intensity of each sample were measured under 405 nm light excitation.

**NOTE:** PL intensity of DAPI rapidly decreases by 30% after 30 min under the light irradiation. However, the decreasing rate in PL intensity of the UA-SiQD is low compared to that of DAPI. The high resistance of SiQD to the 405-nm light irradiation suggests the potential to realize the multimodal fluorescence imaging.


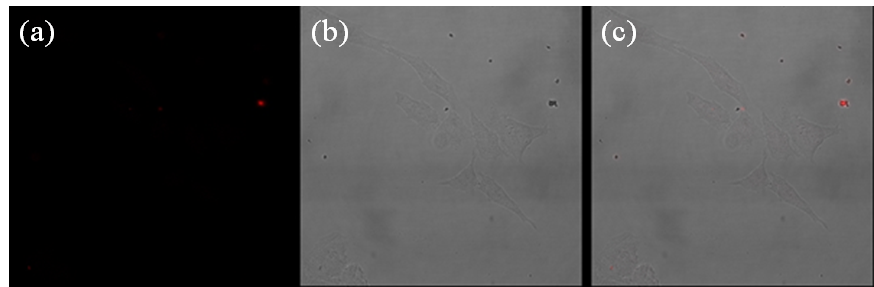


Figure S6. Confocal microscopic images of HeLa cells incubated for 1 hr at 4°C with SiQDs of 11%-UA coverage: (a) Differential interference contrast (DIC), (b) fluorescence and (c) overlap images.
